# Epigenetic Activation of *BRCA1* by Genistein In Vivo and Triple Negative Breast Cancer Cells Linked to Antagonism toward Aryl Hydrocarbon Receptor

**DOI:** 10.3390/nu11112559

**Published:** 2019-10-23

**Authors:** Micah G. Donovan, Ornella I. Selmin, Thomas C. Doetschman, Donato F. Romagnolo

**Affiliations:** 1 Cancer Biology Graduate Interdisciplinary Program, The University of Arizona, Tucson, AZ 85724, USA; 2 The University of Arizona Cancer Center, The University of Arizona, Tucson, AZ 85724, USA; selmin@email.arizona.edu (O.I.S.); tdoetsch@email.arizona.edu (T.C.D.); 3 Department of Nutritional Sciences, The University of Arizona, Tucson, AZ 85721, USA; 4 Department of Cellular and Molecular Medicine, The University of Arizona, Tucson, AZ 85724, USA

**Keywords:** BRCA1, epigenetics, aryl hydrocarbon receptor, DNA methylation, genistein, breast cancer

## Abstract

Triple negative breast cancers (TNBC) are the most aggressive and lethal breast cancers (BC). The aryl hydrocarbon receptor (AHR) is often overexpressed in TNBC, and its activation results in the epigenetic silencing of *BRCA1*, which is a necessary factor for the transcriptional activation of estrogen receptor (ER)α. The dietary isoflavone genistein (GEN) modulates *BRCA1* CpG methylation in BC cells. The purpose of this study was to investigate the effect of GEN on *BRCA1* epigenetic regulation and AHR activity in vivo and TNBC cells. Mice were administered a control or GEN-enriched (4 and 10 ppm) diet from gestation through post-natal day 50. Mammary tissue was analyzed for changes in *BRCA1* regulation and AhR activity. TNBC cells with constitutively hypermethylated *BRCA1* (HCC38) and MCF7 cells were used. Protein levels and mRNA expression were measured by Western blot and real-time PCR, respectively. *BRCA1* promoter occupancy and CpG methylation were analyzed by chromatin immunoprecipitation and methylation-specific PCR, respectively. Cell viability was determined by 3-(4,5-dimethylthiazol-2-yl)-2,5-diphenyltetrazolium bromide (MTT) assay. GEN administered in the diet dose-dependently decreased basal *Brca1* methylation and AHR activity in the mammary gland of adult mice. HCC38 cells were found to overexpress constitutively active AHR in parallel with *BRCA1* hypermethylation. The treatment of HCC38 cells with GEN upregulated BRCA1 protein levels, which was attributable to decreased CpG methylation and AHR binding at *BRCA1* exon 1a. In MCF7 cells, GEN prevented the 2,3,7,8-tetrachlorodibenzo-p-dioxin (TCDD)-dependent localization of AHR at the *BRCA1* gene. These effects were consistent with those elicited by control AHR antagonists galangin (GAL), CH-223191, and α-naphthoflavone. The pre-treatment with GEN sensitized HCC38 cells to the antiproliferative effects of 4-hydroxytamoxifen. We conclude that the dietary compound GEN may be effective for the prevention and reversal of AHR-dependent *BRCA1* hypermethylation, and the restoration of ERα-mediated response, thus imparting the sensitivity of TNBC to antiestrogen therapy.

## 1. Introduction

Breast cancers (BC) are the most common malignancies and causes of cancer mortality in women worldwide [1]. Triple negative breast cancers (TNBC) account for ~15–20% of BC cases and refer to a heterogenous group of BC that lacks expression of estrogen receptor (ER)α, progesterone receptor (PR), and human epidermal growth factor receptor 2 (HER2) [2]. Systemic chemotherapy is the main treatment option for TNBC, since these tumors lack molecular targets for therapy. Unfortunately, only ~20–30% of TNBC patients have a pathological complete response to neoadjuvant therapy [3]. Therefore, key research priorities related to the management of TNBC include (1) deriving methods for effective prevention, and (2) identifying compounds for use in the adjuvant setting to enhance therapeutic response.

The *BRCA1* gene encodes a 220-kDa nuclear phosphoprotein (BRCA1) that functions as a tumor suppressor through involvement in DNA damage repair, cell cycle control, transcriptional regulation, apoptosis, and mRNA splicing [4]. Women who inherit *BRCA1* mutations have a ~72% lifetime risk of developing BC [5], the majority of which are TNBC [6]. Similar to *BRCA1* mutation carriers, the hypermethylation of *BRCA1* is associated with a BRCA1-deficient phenotype (i.e., BRCAness) [7] and increased odds of developing sporadic breast tumors that are TNBC [8]. The hypermethylation of *BRCA1* is reported in ~20–65% of sporadic TNBC [9,10,11] and contributes to the biallelic inactivation of functional alleles in tumors from *BRCA1* mutation carriers [12,13,14].

Dietary factors are considered to play a key role in both the prevention and progression of BC [15]. The consumption of genistein [GEN (i.e., 5,7-dihydroxy-3-(4-hydroxyphenyl)chromen-4-one)], a non-toxic naturally occurring isoflavone found in soy, has been suggested to lower rates of BC in Eastern Asian countries [16,17]. Epidemiological studies indicated that BC risk may be decreased by ~40% with higher consumption of soy and soy isoflavones [18,19,20,21,22,23,24]. The results of mechanistic studies in vitro show that the anti-tumorigenic activity of GEN in BC cells is largely attributable to the preferential induction of ERβ, which suppresses ERα signaling [25]. In ERα-positive MCF-7 cells, the overexpression of ERβ enhances the antiproliferative effects of GEN [26]. Other anti-tumorigenic effects of GEN in BC cells include inhibition of protein tyrosine kinase [e.g., epidermal growth factor receptor (EGFR), platelet-derived growth factor receptor (PDGFR)] and nuclear factor kappa-light-chain-enhancer of activated B cells (NF-κB) signaling [25]. In addition, GEN induces cell cycle arrest in the G2/M phase of the cell cycle [27] and inhibits DNA methyltransferase (DNMT) activity [28,29,30,31] in TNBC cells.

Dietary studies in rodents demonstrate that the dose and timing of exposure to GEN influence tumor response. For example, soy isoflavones administered during the perinatal (i.e., gestation and lactation) or postweaning (4 weeks of age onward) periods increase respectively tumor burden and the onset of mammary adenocarcinoma development in mouse mammary tumor virus (MMTV)-neu transgenic mice [32,33]. On the other hand, GEN administered before puberty appears to delay mammary tumor development in rats [34] and decrease 7,12-dimethylbenz[a]anthracene (DMBA)-induced mammary tumor incidence and aggressiveness in mice [35]. Interestingly, the anti-mammary tumor effects of GEN are not observed in *Brca1*^+/−^ mice, possibly suggesting a requirement for coincident *Brca1* expression.

The *BRCA1* promoter harbors multiple cognate binding sequences for the aryl hydrocarbon receptor (AHR), which are referred to as xenobiotic response elements (XRE) [36]. The AHR is a highly conserved ligand-activated transcription factor of the basic helix–loop–helix–PER–ARNT–SIM (bHLH/PAS) family [37]. It regulates a gene battery involved in the metabolism and conjugation of steroids, drugs, and other xenobiotics [38]. Under normal conditions, *BRCA1* is induced by estradiol (E2) through a non-canonical mechanism of ERα, whereby an activator protein (AP)/ERα transcription complex containing the unliganded AHR assembles at the proximal *BRCA1* promoter [39]. Upon activation with exogenous ligands [e.g., 2,3,7,8-tetrachlorodibenzene(p)dioxin (TCDD)], the AHR colocalizes at the *BRCA1* promoter with DNMT3a, DNMT3b, and DNMT1 [36,40,41]. This leads to hypermethylation at a cytosine-guanine dinucleotide (CpG) island proximal to exon 1a of *BRCA1* and the suppression of E2-dependent *BRCA1* transactivation.

We recently reported that GEN prevents *BRCA1* hypermethylation in ERα-positive MCF7 BC cells treated with the AHR agonist TCDD, and reverses constitutive *BRCA1* CpG methylation in ERα-negative HER2-enriched cells with constitutive high levels of AHR [42]. Due to the association between the TNBC phenotype and increased *AHR* expression and *BRCA1* CpG methylation, we hypothesized that GEN counteracts the AHR-dependent repression of *BRCA1*. We show that a GEN-enriched diet administered to mice from conception through lactation, weaning, and adult life decreases basal *Brca1* methylation and AHR activity in the adult mammary gland. We also document that in HCC38 TNBC cultured cells, the AHR is overexpressed and constitutively active. Conversely, the treatment of HCC38 cells with GEN and selected AHR antagonists increases BRCA1 protein levels via CpG demethylation and decreased recruitment of the AHR at the *BRCA1* promoter. The latter effects are observed in parallel with increased ERα expression, leading to the sensitization of HCC38 TNBC cells to the growth inhibitory effects of 4-hydroxytamoxifen (4-OHT).

## 2. Materials and Methods

### 2.1. Animal Models

BRCA1^F22/24^ mice (The Jackson Laboratory, stock no. 017835) were bred and maintained in the Genetically Engineered Mouse Model (GEMM) Core under protocol #15-055 approved on 15 August 2018 by the Institutional Animal Care and Use Committee (IACUC) program of The University of Arizona (PHS Assurance #D16-00159, USDA Reg.#86-R-0003). Sires and dam were administered diets containing 0, 4, or 10 ppm GEN starting when breeding pairs were set up, to ensure the in utero exposure of offspring to GEN. Animals were allowed chow and water ad libitum. At birth, female offspring nursed from their biological mothers, which were continued on experimental diets to provide lactational exposure to control diet or diet supplemented with GEN. Offspring were weaned onto the same diet, which was administered until sacrifice at post-natal day 50 (PND50). At the time of sacrifice, mice were euthanized by CO_2_ asphyxiation. Mammary glands were surgically removed, immediately snap frozen in liquid nitrogen, and then stored at −80 °C for later use.

### 2.2. Cell Culture and Reagents

MCF7, UACC3199, and HCC38 BC cells were obtained from the American Type Culture Collection (ATCC). Cells were cultured as previously described [42]. Briefly, MC7 cells were maintained in Dulbecco’s Modified Eagle’s Medium/Ham’s F-12 50/50 (DMEM, Corning, Ref. 10-090-CV) supplemented with 10% fetal bovine serum (FBS, Peak Serum, Ref. PS-FBS). UACC3199 and HCC38 cells were maintained in Roswell Park Memorial Institute (RPMI) 1640 medium (Corning, Ref. 10-040-CV) supplemented with 10% FBS. Cells were maintained at 37 °C with 5% CO_2_ and ~85% relative humidity. All experiments were conducted in phenol red-free medium supplemented with charcoal stripped FBS (Ref. F6765) in 100 × 15 mm petri dishes (Falcon, Ref. 351029). Phenol red-free DMEM (Ref. 21041) and RPMI (Ref. 11835) were from Gibco. GEN was purchased from ChemCruz (Ref. sc-3515) and estradiol (E2, Ref. E8875), α-naphthoflavone (NF, Ref. N-5757), galangin (GAL, Ref. 282200), CH-223191 (Ref. C8124), and 4-OHT (Ref. H7904) were purchased from Sigma-Aldrich. TCDD was supplied by the National Cancer Institute, Division of Cancer Biology, Chemical and Physical Carcinogenesis Branch (NIH), and distributed by Midwest Research Institute (contracts 64 CFR 72090 and 64 CFR 28205). GEN, GAL, and NF were solubilized in ethanol (EtOH) and 4-OHT, CH-223191, and TCDD were solubilized in DMSO. EtOH and DMSO were added to cell media as vehicle (VEH)-treated controls were indicated. HCC38 cells were seeded for experiments at a density of ~2.5 × 10^5^ cells/dish. MCF7 and UACC3199 cells were seeded at a density of 5 × 10^5^ cells/dish. Cells were seeded in complete media, which was changed to phenol-red free medium after 24 h as previously described [42]. Treatments occurred after 72 h equilibration in phenol-red free medium. For experiments in MCF7 cells treated with TCDD, cells were pretreated for 12 h with TCDD alone or in combination with GEN or GAL before treatment with E2. At harvest, cells were washed twice with ice-cold PBS and then scraped for collection. Cell suspensions were centrifuged at 300× *g* for 5 min, and cell pellets were stored at −20° or used immediately for analysis.

### 2.3. Western Blotting

Western blots were performed as previously described [42]. Briefly, whole-cell protein lysates were prepared by incubating cell culture pellets or mammary gland tissue (~30 mg) in Pierce RIPA Buffer (Thermo Fisher Scientific, Waltham, MA, USA; Ref. 89901) supplemented with 1% protease inhibitor cocktail (VWR, Ref. M250) on ice for 30 min with periodic vortexing. Protein concentration was determined using the Nanodrop1000 Spectrophotometer (Thermo Fisher Scientific, Waltham, MA, USA). Before polyacrylamide electrophoresis (PAGE), normalized samples containing 100 μg of protein lysate were heated in a block at 65 °C for 4 min; then, an equal volume of Lamelli buffer (Biorad, Hercules, CA, USA; Ref. 161-0737) with 1% β-mercaptoethanol was added, and the mix was boiled for 4 min. Then, samples were cooled to room temperature and centrifuged at maximum speed for 30 s. Proteins were separated by PAGE using Novex Wedgewell 4–12% tris-glycine gels (Invitrogen, Carlsbad, CA, USA; Ref. XP04120BOX) with a constant voltage of 100 V for ~75 min. Proteins were transferred to Protran 0.2-μm nitrocellulose blotting membranes (Amersham, Little Chalfont, UK; Ref. 10600001) using the Mini Blot Module (Ref. B1000) and Mini Gel Tank (Ref. A25977) wet-transfer system by Invitrogen. Transfer occurred at a constant 15 V in tris-glycine transfer buffer with 15% methanol. Blocking was performed with 5% milk in tris buffered saline (TBS) for 1 h at room temperature. Immunoblotting was carried out with antibodies raised against BRCA1 (Boster Bio, Pleasanton, CA, USA; Ref. PB9015), ERα (Santa Cruz, Ref. MC-20), AHR (Santa Cruz Biotechnology Inc, Dallas, TX, USA; Ref. B-11), and glyceraldehyde 3-phosphate dehydrogenase (GAPDH) (Origene, Rockville, MD, USA; Ref. TA890003). Primary antibodies were diluted in TBS-T + 2% milk, and membranes were incubated overnight at 4 °C. Following incubation with a primary antibody, membranes were incubated in a secondary antibody (diluted in TBS-T + 2% milk) for 1 h at room temperature. Immunocomplexes were detected by enhanced chemiluminescence (GE Healthcare Life Sciences, Chicago, IL; USA) or near-infrared scanning using an Odyssey CLx (Li-COR, Lincoln, NE, USA). Immunocomplexes of GAPDH served as an internal control for equal sample loading. Densitometry was performed using ImageJ software (NIH).

### 2.4. mRNA Analyses

mRNA was extracted from mouse mammary glands and cell pellets using the Quick-RNA MiniPrep kit from Zymo as per the manufacturer’s instructions (Zymo, Irvine, CA, USA; Ref. 11-328). Briefly, cell culture pellets or ~30 mg mammary tissue were suspended in RNA lysis buffer and sonicated on ice for 4 pulses of 10 s each. DNA was digested using DNase I, and RNA samples were eluted using RNase-free water. Purified RNA was stored at −80 °C or used immediately for cDNA synthesis. cDNA was prepared using a qScript cDNA Synthesis Kit as per the manufacturer’s recommended protocol (Quantabio, Beverly, MA, USA; Ref. 95047-025). Purified cDNA was stored at −20 °C or used immediately in real-time qPCR assays, as previously described [42]. The qPCR was carried out in a 20-μL volume with a master mix consisting of 10 μL of PerfeCta SYBR Green FastMix with carboxyrhodamine (ROX) (Quantabio), 2 μL of forward and reverse primers, 4 μL of RNase-free water, and 2 μL of cDNA template. Reaction parameters for PCR were: 95 °C × 10 min (escalate by 1.6 °C/s), followed by 40 cycles of 95 °C × 15 s, and then an annealing temperature × 1 min. Relative quantities (RQ) of mRNA were determined using the 2^−ΔΔCT^ method [43] using *GAPDH* mRNA as an internal standard. Human primer sequences (designed by Sigma Aldrich, St. Louis, MO, USA) were: *AHR*: forward, 5′-GAAGCCGGTGCAGAAAACAG-3′, reverse: 5′-GCCGCTTGGAAGGATTTGAC-3′; *CYP1B1:* forward, 5′-AACGTCATGAGTGCCGTGTGT-3′, reverse, 5′-GGCCGGTACGTTCTCCAAATC-3′; *CYP1A1:* forward 5′-TAACATCGTCTTGGACCTCTTTG-3′, reverse, 5′-GTCGATAGCACCATCAGGGGT-3′; and *GAPDH:* forward, 5′-ACCCACTCCTCCACCTTT-3′, reverse, 5′-CTCTTGTGCTCTTGCTGGG-3′. Mouse primer sequences were: *Cyp1b1*: forward, 5′-TCTTTACCAGATACCCGGATG-3′, reverse 5′-CACAACCTGGTCCAACTCAG-3′; and *Gapdh:* forward, 5′-CACTTGAAGGGTGGAGCCAA-3′, reverse, 5′-AGTGATGGCATGGACTGTGG-3′.

### 2.5. DNA Methylation

CpG methylation was determined by sodium bisulfite conversion of gDNA followed by real-time PCR [quantitative methylation-specific PCR (qMSP)], as previously described [42]. Genomic DNA (gDNA) from mouse mammary glands and cell pellets was isolated with the DNeasy Blood and Tissue kit using the manufacturer’s instructions (Qiagen, Hilden, Germany; Ref. 69506). Bisulfonation was performed using the EZ DNA Methylation-Gold™ Kit (Zymo, Ref. D5005). gDNA samples were normalized to a concentration of 500 ng/20 μL with RNase-free water and incubated with sodium bisulfite solution in a thermal cycler with the following cycle parameters: 98 °C for 10 min, 64 °C × 2.5 h, and 4 °C for holding. Following bisulfite conversion, purified bisulfonated DNA (bsDNA) was stored at −20 °C or used immediately in qPCR with the following master mix: 10 μL of PerfeCta SYBR Green FastMix with ROX (Quantabio), 2 μL of forward and reverse primers, 4 μL of RNase-free water, and 2 μL of bsDNA template. Data are expressed as the ratio of methylated over unmethylated amplicons. Human primer sequences for methylation-specific primers were: *mBRCA1:* forward, 5′-CGGTAGTTTTTGGTTTTCGTGG-3′, reverse, 5′-ATCTCAACGAACTCACGCCG-3′; *umBRCA1:* forward, 5′-TTGGTTTTTGTGGTAATGGAAAAGTGT-3′, reverse, 5′-CAAAAAATCTCAACAAACTCACACCA-3′; *mESR1:* forward, 5′-GGTTTTTGAGTTTTTTGTTTTG-3′, reverse, 5′-AACTTACTACTATCCAAATACACCTC-3′; *umESR1*: forward, 5′-GGATATGGTTTGTATTTTGTTTGT-3′, reverse, 5′-ACAAACAATTCAAAAACTCCAACT-3′. Primer sequences for mouse MSP primers were: *mBrca1:* forward, 5′-GTTAGCGTTAGGCGTTAAGC-3′, reverse, 5′-AAAACTATCTCACCTTTTCTCCGA-3′; and *umBrca1:* forward, 5′-AAAGGTTAGTGTTAGGTGTTAAGTGG-3′, reverse, 5′- AAAACTATCTCACCTTTTCTCCAAA-3′.

### 2.6. Chromatin Immunoprecipitation

Chromatin immunoprecipitation (ChIP) assays were performed using the Pierce Magnetic ChIP kit as per the manufacturer’s instructions (Thermo Fisher Scientific, Ref. 26157). Chromatin was immunoprecipitated with antibodies raised against AHR (Santa Cruz Biotechnology Inc, Ref. B-11) overnight at 4 °C. Pulldown was performed using ChIP-grade protein A/G magnetic beads (Thermo Fisher Scientific, Ref. 26162) and a DynaMag-2 (Invitrogen, Ref. 12321D). Purified DNA was stored at −20 °C or used immediately in real-time PCR. The master mix for qPCR contained the following: 10 μL of PerfeCta SYBR Green FastMix with ROX (Quantabio), 2 μL of forward and reverse primers, 3 μL of RNase-free water, and 3 μL of cDNA template. Primer sequences for *BRCA1* exon 1a were: forward, 5′-CTCCCATCCTCTGATTGTACCTTGAT-3′ and reverse, 5′-CAGGAAGTCTCAGCGAGCTCAC-3′.

### 2.7. Cell Viability Assay

Cell viability assays were carried out as previously described using the 3-(4,5-dimethylthiazol-2-yl)-2,5-diphenyltetrazolium bromide (MTT) colorimetric assay [42]. Cells were seeded in 48-well plates at a density of ~1000 cells/well. After 24 h, cells were put in untreated phenol red-free media for 3 days (d). After equilibration, cells were pretreated with either EtOH, GEN, or GAL for 4 d. Then, cells were treated with E2 alone or in combination with 4-OHT for 72 h. After treatment, 30 μL of MTT dye (5 mg/ml in PBS) was added directly to the culture media, and cells were incubated at 37 °C for 4 h. The media/MTT mixture was aspirated, and formazan crystals were solubilized by DMSO for 1 h at room temperature. After solubilization, the absorbance was read at 570 and 650 nm using a Synergy HT plate reader (Bio-Tek Instruments, Winooski, VT, USA). Relative absorbance was determined by subtracting the 650-nm values from the 570-nm values, and the results were normalized to E2-treated sample absorbance.

### 2.8. Statistical Analysis

Comparisons between two sample means were conducted by Student’s t-test, and significance was determined to be *p* < 0.05. For multiple comparisons (i.e., >2), statistical analyses were executed by one-way ANOVA. Post hoc multiple comparisons between means were performed using Tukey’s honestly significant difference (HSD) test after the main effects and interactions were found to be significant at *p* < 0.05. Data are presented as mean values ± SEM. Means and SEM values were determined for cell culture experiments using ≥3 biological replicates from individual experiments. Means and SEM for analyses of mouse tissue were determined using ≥7 biological samples from individual animals. In the figures, significant differences are denoted with asterisks (*, *p* < 0.05; **, *p* < 0.01; ***, *p* < 0.001)) or different letters (i.e., a > b > c, etc.).

## 3. Results

### 3.1. Lifetime Exposure to Dietary GEN Decreases Basal Brca1 Methylation in Mouse Mammary Tissue of Offspring

**Figure 1:**
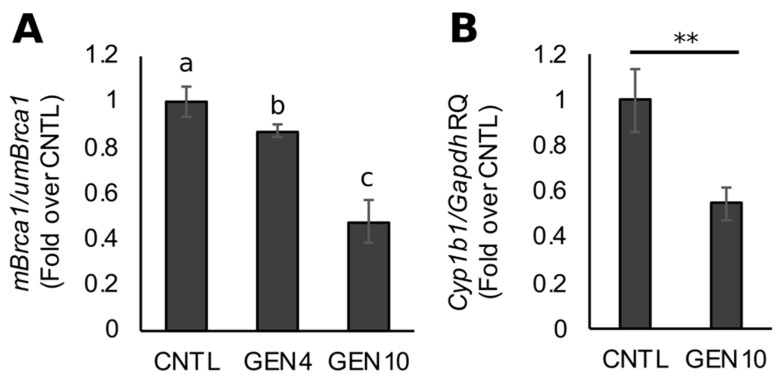
Lifetime exposure to genistein (GEN) decreases basal *Brca1* cytosine-guanine dinucleotide (CpG) methylation and aryl hydrocarbon receptor (AHR) activity in mouse offspring mammary tissue. Mice were exposed to diets containing 0, 4, or 10 ppm GEN starting at conception and through nursing, weaning, and adulthood. (**A**) *Brca1 CpG* methylation in a mammary gland of post-natal day 50 (PND50) female offspring. Bars represent the mean ratios of methylated *Brca1* to unmethylated *Brca1* amplicons ± SEM from ≥7 individual animals. (**B**) *Cyp1b1* mRNA expression in PND50 mammary glands. Bars represent mean RQ ± SEM from ≥7 individual animals. Means with different letters (a > b > c) or asterisks (**, *p* < 0.01) indicate statistical significance (*p* < 0.05).

We recently reported on the capacity for GEN to counteract AHR-dependent *BRCA1* CpG methylation in ERα-positive MCF7 and ERα-negative/HER2-enriched UACC3199 cells [42]. To study the in vivo effects of GEN on *BRCA1* epigenetic regulation and AHR activity in mammary gland, we administered diets containing 0 (CNTL), 4 (GEN4), or 10 (GEN10) ppm GEN to mice over the lifetime (i.e., during gestation, lactation, and after weaning). Compared to CNTL mice offspring, the relative ratio of methylated to unmethylated *Brca1* amplicons (*mBrca1*/*umBrca1*) was decreased by ~15% (*p* = 0.0079) and ~50% (*p* = 0.0034) in the mammary glands of female mice offspring fed a GEN4 and GEN10 diet, respectively (Figure 1A). Given the magnitude of the CpG methylation effect in the GEN10 offspring, we investigated *Cyp1b1* expression as a surrogate biomarker for AHR activity. Compared with CNTL mice, mammary gland expression of *Cyp1b1* was on average ~50% lower in GEN10 female offspring (*p* = 0.007911, Figure 1B). These data suggested that GEN exerted antagonistic effects on basal AHR-dependent epigenetic regulation of *BRCA1* in vivo.

### 3.2. BRCA1 CpG Hypermethylation Associates with Constitutively Active AHR in TNBC Cells

**Figure 2:**
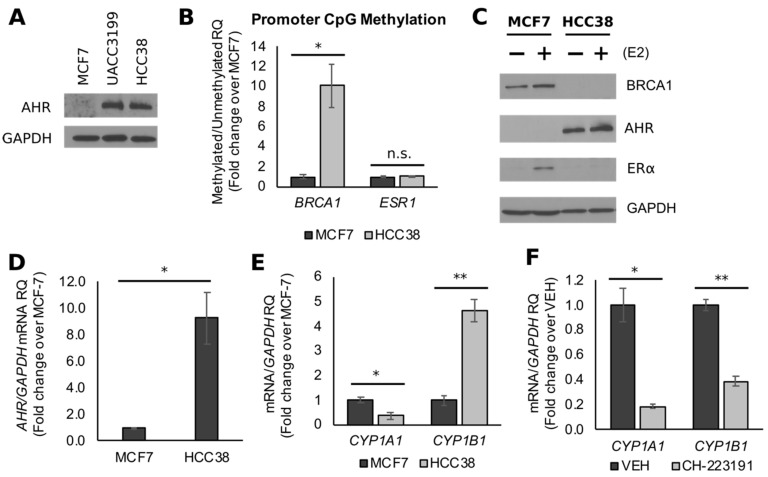
*BRCA1* CpG hypermethylation associates with constitutively active AHR in triple negative breast cancers (TNBC) cells. (**A**) Representative Western blot images comparing immunocomplexes of AHR and internal standard glyceraldehyde 3-phosphate dehydrogenase (GAPDH) in HCC38 and UACC3199 cells. (**B**) Data from methylation-specific PCR (MSP) comparing *BRCA1* and *ESR1* CpG methylation in HCC38 and MCF7 cells. (**C**) Bands represent immunocomplexes for BRCA1, estrogen receptor (ER)α, AHR, and internal standard GAPDH in MCF7 and HCC38 cells. (**D**) Comparison of *AHR* mRNA expression in HCC38 and MCF7 cells. (**E**) Expression of AHR targets *CYP1A1* and *CYP1B1* in HCC38 and MCF7 cells. (**F**) *CYP1A1* and *CYP1B1* expression in HCC38 cells treated with the AHR antagonist CH-223191. Bars represent sample means ± SEM from ≥3 biological replicates from individual experiments. VEH: vehicle-treated control. Asterisks indicate significant differences (*, *p* < 0.05; **, *p* < 0.01).

Given the link between *BRCA1* hypermethylation and TNBC phenotype, we compared the effects of GEN on *BRCA1* CpG methylation and AHR activity in HCC38 and UACC3199 cells. The UACC3199 cell line is ER/PR-negative, but expresses HER2, whereas HCC38 cells form a model of TNBC. However, the two lines share a similar phenotype in regard to *BRCA1* hypermethylation [44,45] and AHR overexpression (Figure 2A). Previous studies showed that CpG methylation of the *BRCA1* promoter in HCC38 cells was associated with low levels of BRCA1 protein [45]. Compared to MCF7 cells, we found by MSP that the relative ratio of *mBRCA1*/*umBRCA1* was on average ~10-fold higher (*p* = 0.0158) in HCC38 cell cultures (Figure 2B). Western blot measurements confirmed the E2-dependent upregulation of BRCA1 in MCF7 cells, which served as a positive control for the detection of BRCA1 immunocomplexes throughout the study (Figure 2C). In contrast, BRCA1 protein levels were lower in HCC38 cells both in the presence and absence of E2 (Figure 2C). *ESR1* promoter methylation was not significantly different (*p* = 0.1995) between the two cell lines (Figure 2B), despite the limited detection of ERα in HCC38 cells (Figure 2B). These results suggest that different epigenetic mechanisms other than CpG methylation may contribute to the reduction of ERα expression in HCC38 cells.

Activation of the AHR leads to CpG methylation of the *BRCA1* promoter [40,41], and the overexpression of *AHR* mRNA is observed in parallel with *BRCA1* hypermethylation in TNBC patients [46]. Moreover, rodent and human mammary tumors overexpress constitutively active AHR, which is characterized by elevated *CYP1B1* but not *CYP1A1* mRNA [47]. In HCC38 cells, the AHR protein was markedly increased (Figure 2C), and *AHR* mRNA levels were on average ~9.0-fold higher (*p* = 0.0155) compared to MCF7 cells (Figure 2D). The expression of *CYP1A1* mRNA was lower ~60% (*p* = 0.04), whereas *CYP1B1* expression was ~4.7-fold higher (*p* = 0.017) in HCC38 cells compared to MCF7 cells (Figure 2E). The treatment of HCC38 cells with the AHR antagonist CH-223191 significantly decreased the expression of *CYP1A1* and *CYP1B1* by ~80% (*p* = 0.015) and ~60% (*p* = 0.021), respectively (Figure 2F). These data suggest that CpG hypermethylation of *BRCA1* associates with overexpressed and constitutively active AHR protein in a model of TNBC cells (HCC38).

### 3.3. GEN Rescues BRCA1 Protein Levels in TNBC Cells with CpG Hypermethylated BRCA1

**Figure 3:**
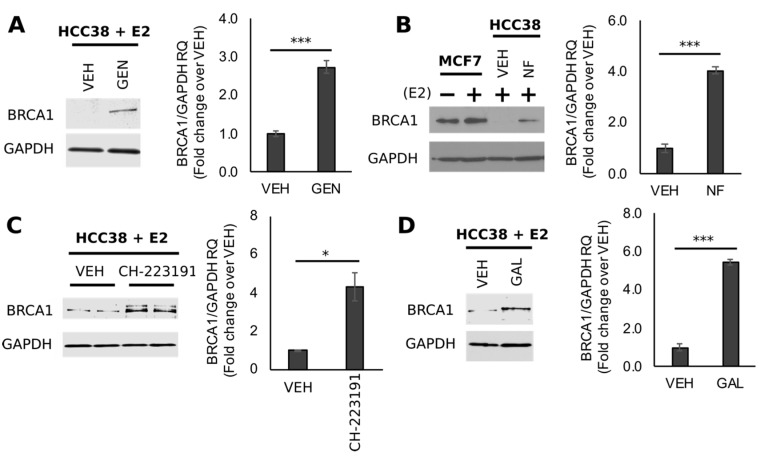
Genistein rescues BRCA1 protein levels in TNBC cells with CpG hypermethylated *BRCA1*. Bands are immunocomplexes of BRCA1 and internal standard GAPDH in HCC38 cells treated with (**A**) GEN, (**B**) α-naphthoflavone (NF), (**C**) CH-223191, and (**D**) galangin (GAL). Images are representative blots from ≥3 individual experiments. For densitometry histograms, bars represent mean RQ ± SEM from ≥3 biological replicates from individual experiments. Asterisks indicate significant differences (*, *p* < 0.05; ***, *p* < 0.001).

In a recent cell culture study, we documented that GEN at the concentration of 10 μM demethylated *BRCA1* in HER2-enriched UACC3199 cells [42]. To determine if GEN exerted similar CpG demethylating effects on *BRCA1* in TNBC, we treated HCC38 cells with GEN (10 μM) and various AHR antagonists in the presence of E2 (10 nM). Results of Western blots indicated that GEN (Figure 3A) increased BRCA1 protein levels ~2.7-fold (*p* < 0.0001) compared to cells treated with E2 alone. Similar stimulatory effects were observed with protein lysates from HCC38 cells treated with the AHR antagonists NF (2 μM, Figure 3B), CH-223191 (10 μM, Figure 3C), and GAL (2 μM, Figure 3D), which increased BRCA1 protein by ~4.0-fold (*p* = 0.0002), ~4.3-fold (*p* = 0.0433), and ~5.4-fold (*p* < 0.0001), respectively. Since NF and GAL share similar binding affinity for the AHR [48], an equimolar concentration (2 μM) for these compounds was adopted for subsequent cell culture studies. The concentration of CH-223191 used here (10 μM) was based on previous investigations demonstrating antagonism toward the AHR in BC cells [49].

### 3.4. Rescue of BRCA1 Expression Is Linked to CpG Demethylation and AHR Inhibition in TNBC Cells

**Figure 4:**
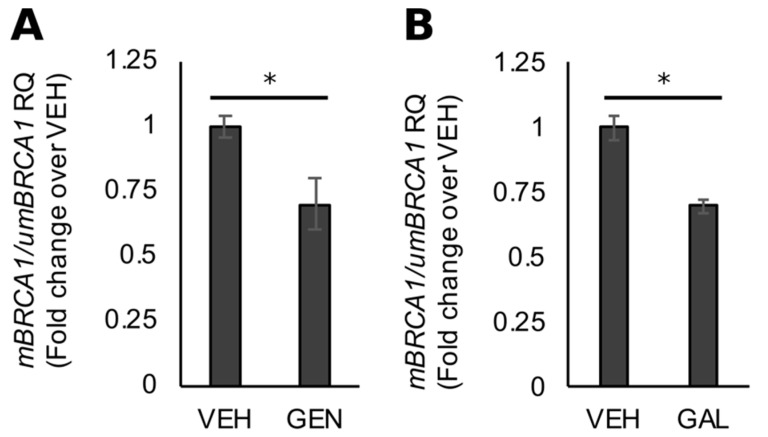
Rescue of *BRCA1* expression is linked to CpG demethylation in TNBC cells. The average ratio of *mBRCA1*/*umBRCA1* in HCC38 cells treated for 96 h with (**A**) GEN or (**B**) GAL. Bars represent sample means ± SEM from ≥3 biological replicates from individual experiments. Asterisks denote significance (*, *p* < 0.05).

Consistent with our work with UACC3199 cells [42], the increase in BRCA1 protein observed with GEN treatment in HCC38 cells was associated on average with an ~30% decrease (*p* = 0.05) in CpG methylation at the *BRCA1* promoter (Figure 4A). As a control, we determined that the stimulatory effects of GAL on BRCA1 protein levels were also associated a with a decrease (~25%) in *BRCA1* promoter methylation (Figure 4B, *p* = 0.02), based on the information that GEN is an antagonist of AHR transcriptional activity in colon cancer cells [50] and a dual agonist/antagonist in ER + BC cells [51].

**Figure 5:**
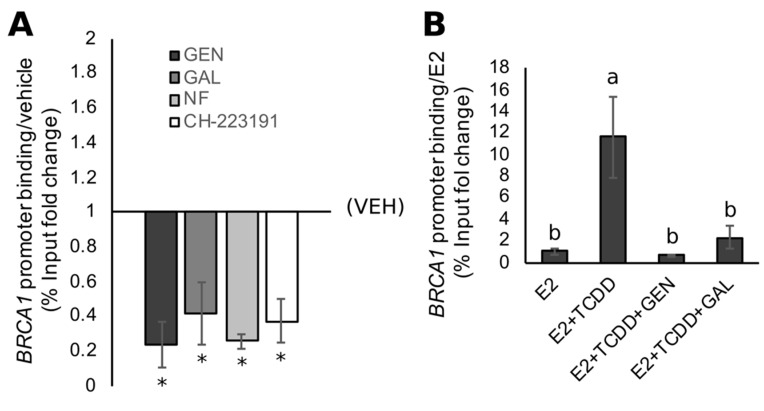
Antagonism toward AHR binding at the *BRCA1* promoter by GEN. Results show the relative binding of AHR at the *BRCA1* promoter from chromatin immunoprecipitation (ChIP) assays in (**A**) HCC38 cells treated with GEN and various AHR antagonists; and (**B**) MCF7 cells treated with estradiol (E2) and 2,3,7,8-tetrachlorodibenzo-p-dioxin (TCDD) in the presence or absence of GEN and GAL. Relative binding was determined as the percent input (ChIP/Input*100) from treated samples normalized to VEH (**A**) or E2 (**B**) controls. Bars represent sample means ± SEM from ≥3 biological replicates from individual experiments. Means with different letters (a > b) or asterisks (*) indicate significance (*, *p* < 0.05).

We tested if GEN altered the recruitment of the AHR to the *BRCA1* promoter. We utilized a ChIP assay in which fixed DNA was immunoprecipitated with an AHR antibody, and the resulting purified DNA was amplified in PCR reactions using primers spanning exon 1a of the *BRCA1* promoter. Compared to control cells, the treatment with GEN reduced AHR binding (~74%) at the exon 1a of *BRCA1* (Figure 5A). The latter effect was similar in magnitude to the one elicited by the control compounds GAL (~75%), NF (~74%), and CH-223191 (~63%).

Previously, we reported that the treatment of MCF7 cells with TCDD induces AHR recruitment to the *BRCA1* promoter, leading to the repression of E2-stimulated *BRCA1* transactivation [41]. To test if the antagonistic effects of GEN on AHR binding at the *BRCA1* gene was limited to constitutively active AHR, we cotreated MCF7 cells with E2 and TCDD in the presence and absence of GEN. Compared with cells treated with E2 alone, cells treated with the E2/TCDD combination had an ~11-fold increase in AHR binding at the *BRCA1* promoter (Figure 5B). However, the addition of GEN to the E2/TCDD treatment attenuated AHR binding at the *BRCA1* gene by 90% to levels not statistically different from those measured in E2-treated cells. Similar repressive effects (~70%) on AHR recruitment to the *BRCA1* gene were observed upon cotreatment with GAL (Figure 5B).

### 3.5. Genistein Upregulates ER and Sensitizes TNBC Cells to 4-OHT

**Figure 6:**
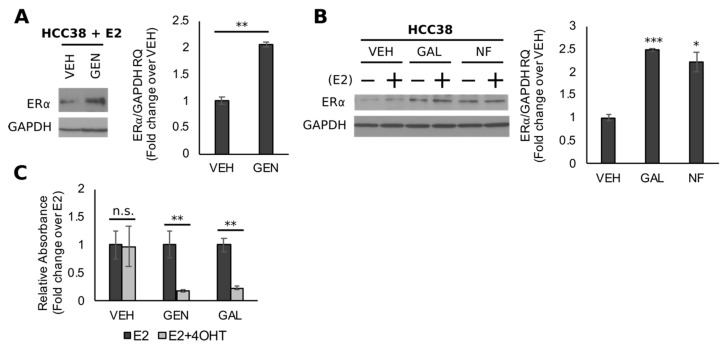
Genistein upregulates ERα expression and sensitizes TNBC cells to 4-OHT. Bands represent immunocomplexes for ERα and internal standard GAPDH in HCC38 cells treated with (**A**) GEN, (**B**) NF, or GAL. Images are representative blots from ≥3 individual experiments. Blots are shown with respective quantitation by densitometry. Bars represent sample means ± SEM from ≥3 biological replicates from individual experiments (**C**) Results from 3-(4,5-dimethylthiazol-2-yl)-2,5-diphenyltetrazolium bromide (MTT) assay in HCC38 cells pre-treated with GEN. Bars represent mean relative absorbance at 570 nm ± SEM from 3 (*n* = 3) independent experiments. Asterisks denote significance (*, *p* < 0.05; **, *p* < 0.01; ***, *p* < 0.001).

BRCA1 is required for the transactivation of *ESR1*, the gene encoding for ERα, and studies have demonstrated that the exogenous expression of functional BRCA1 into *BRCA1*-mutated/ER-negative BC cells (HCC1937) significantly increases ERα protein levels [52]. Moreover, the BRCA1-dependent upregulation of ERα levels sensitizes HCC1937 cells to the growth inhibitory effects of antiestrogens. In line with these findings and our previous studies with UACC3199 cells [42], BRCA1 upregulation by GEN was observed in parallel with increased levels (~2.0-fold, *p* = 0.0033) of ERα (Figure 6A). Comparable effects were seen with the control compounds NF (~2.2-fold, *p* = 0.0232) and GAL (~2.5-fold, *p* = 0.0005) (Figure 6B). Given the growing interest in utilizing non-toxic dietary compounds as adjuvant cancer therapies, we used the MTT cell viability assay to determine if the upregulation of ERα modulated the proliferative response of HCC38 cells to treatment with 4-OHT, which is the active metabolite of tamoxifen. After pre-treatment for 96 h with GEN (HCC38-GEN), GAL (HCC38-GAL), or VEH (HCC38-VEH), cells were treated with E2 alone or in combination with 4-OHT (E2 + 4-OHT) for 72 h. Results from the MTT analysis indicated no difference in cell viability between HCC38-VEH cells treated with E2 or E2 + 4OHT (Figure 6C). In contrast, relative viability was ~75% lower in HCC38 cells treated with GEN (*p* = 0.0028) or GAL (*p* = 0.0010) in combination with E2 + 4-OHT, compared to cells treated with E2 alone (Figure 6C). In summary, the results from our cell culture experiments suggest that GEN reverses CpG hypermethylation of the *BRCA1* promoter in part through the antagonism of constitutively active AHR. Moreover, the GEN-dependent upregulation of BRCA1 increases the expression of ERα, which imparts sensitivity to the growth inhibitory effects of tamoxifen. Taken together, these collective data suggest that GEN counteracts the AHR-dependent epigenetic silencing of *BRCA1* in vivo and in TNBC models. Moreover, the GEN-dependent upregulation of BRCA1 increases the expression of ERα, which may sensitize TNBC cells to antiestrogen therapeutics.

## 4. Discussion

Triple negative breast cancers are clinically aggressive [2], prone to visceral and central nervous system metastasis [53,54], and currently lack targeted therapeutics [55]. The CpG hypermethylation of *BRCA1* is a common epigenetic aberration in sporadic TNBC [9,10,11] and contributes to the silencing of wild-type *BRCA1* alleles in tumors from germline *BRCA1* mutation carriers [12,13,14]. Our group has documented that *AHR* is overexpressed and *BRCA1* is hypermethylated in primary tumors from TNBC patients compared with other BC subtypes and non-malignant tissue [46]. Investigations by other groups have also reported *AHR* overexpression is associated with the TNBC phenotype [56]. High levels of AHR protein are also found in rodent mammary tumors and pre-malignant tissue [46,47,57,58], human BC cell lines [42,47], and primary tumors [59,60]. In normal human mammary epithelial cells, overexpression of the AHR induces malignant transformation [61], whereas AHR knockdown in TNBC cells attenuates tumorigenicity in vitro and in orthotopic mouse models [62]. Our group has characterized the role of AHR activation in the epigenetic silencing of *BRCA1* [36,40,41]. Specifically, we found that GEN prevented *BRCA1* CpG hypermethylation in ERα-positive BC cells treated with an AHR agonist as well as reversed constitutive *BRCA1* CpG methylation in ERα-negative HER2-enriched cells with high levels of AHR [42].

In this study, we first investigated the effect of GEN on the AHR-dependent epigenetic regulation of *BRCA1* in vivo. Dietary GEN is thought to contribute to the lower rates of BC seen in Eastern Asian populations [16,17]. In both rodents and humans, intestinal absorption of GEN occurs rapidly and efficiently due to its small molecular weight (~270 kDa) and lipophilic properties [17]. Studies in rodents have shown the absorption efficiency of total genistein ranges from ~46% to 100%, depending on the animal model, source of GEN, and sex [63,64,65,66,67]. The major pathways of GEN metabolism are glucuronidation and sulfation, and the predominant plasma metabolites are genistein-7-glucuronide-4′-sulfate (G-7G-4′S) and genistein-4′,7-diglucuronide (G-4′,7-diG) [68,69,70]. In mice, after a single oral administration of GEN (20 mg/kg), ~80% was converted to glucuronides or sulfates, whereas ~20% was aglycone GEN [69]. In the present study, we administered GEN-enriched diets (4 and 10 ppm) to breeding pairs, pregnant and lactating mothers, weanlings, and adult offspring. A previous study in female mice administered a GEN-enriched diet (6 ppm) over a similar time course (gestation through lifetime) and found that plasma GEN levels reached ~51.1 nM [33]. In female rats, a 5 ppm GEN diet administered from gestation through the lifetime produced serum levels of ~0.1 and 0.02 μM in adult offspring (PND 140) and weanlings (PND 21), respectively [71]. Here, we show a dose-dependent effect of dietary GEN administered over the entire lifetime on basal *Brca1* CpG methylation in the mammary gland of adult mice. Compared with mice on a control diet (0 ppm GEN), mice fed the low-dose (GEN4) and high-dose (GEN10) diets had ~15% and ~50% less *Brca1* promoter methylation, respectively. This epigenetic effect was linked to the decreased expression of *Cyp1b1* (~50% decreased in GEN10 mice), which confirmed antagonistic effects of GEN toward the AHR. Women from populations with habitual high soy diets are presumed to be exposed to GEN in utero, which has been suggested to reduce BC risk later in life potentially by priming the mammary gland to differentiation [72]. Studies with rodent models have also documented that the protective effect of GEN against mammary tumorigenesis may be dependent on pre-pubertal exposure, particularly starting at conception [72]. Conversely, soy isoflavones (130 ppm) administered in periods that did not comprise the entire lifetime (i.e., not gestational through lifetime) actually increased spontaneous tumor multiplicity and mass in mammary tumor models (i.e., MMTV-neu transgenic mice) [32,33]. Pre-pubertal GEN exposure (500 ppm) was shown to increase *Brca1* expression and decrease DMBA-induced mammary tumor incidence and aggressiveness in mice [35]. However, these effects were not observed in *Brca1*^+/*−*^ mice, possibly suggesting a dependency on *Brca1* expression for the protective effects of GEN against mammary tumorigenesis [35]. Our data suggest that lifetime GEN may decrease BC risk in mice by decreasing basal *Brca1* promoter methylation.

Based on these in vivo results, a second objective of this study was to characterize the effects of GEN in a cell culture model of TNBC with constitutively active AHR. Previously, our group reported that activation of the AHR induced its colocalization at the *BRCA1* promoter with DNMT1, DNMT3a, and DNMT3b, leading to hypermethylation of a CpG island proximal to the *BRCA1* exon 1a [40,41]. Non-quantitative MSP and bisulfite sequencing was used by other investigators to show that CpG methylation at 30/30 CpG sites of the *BRCA1* promoter associated with decreased levels of BRCA1 protein and mRNA in HCC38 cells [45]. Using real-time MSP, we documented here that the ratio of *mBRCA1*/*umBRCA1* was ~10-fold higher in HCC38 compared to MCF7 cells. The *BRCA1* hypermethylation was observed in parallel with the marked upregulation of AHR expression (protein and mRNA). When compared to MCF7 cells, expression of the AHR target gene *CYP1A1* was decreased in HCC38 cells, whereas *CYP1B1* expression was elevated. This trend was in line with previous reports documenting that constitutively active AHR in rodent and human mammary tumors associated with elevated *CYP1B1*, but not *CYP1A1* or mRNA [47]. Earlier studies revealed that AHR overexpression and constitutive activity in TNBC cells was likely due to a positive amplification loop, whereby the AHR-dependent induction of tryptophan 2,3-dioxygenase (TDO2) caused the accumulation of endogenous AHR ligands in the form of tryptophan metabolites (e.g., kynurenine, kynurenic acid) [73]. Alternatively, constitutive AHR expression and activity could be due to the loss of AHR repressor (AHRR) expression, which imparts a negative feedback regulation on AHR signaling and activity [74].

We observed that the treatment of HCC38 cells with GEN and various high-affinity AHR antagonists (NF, CH-223191, GAL) increased cellular levels of the BRCA1 protein. This effect was consistent with the stimulatory effects of GEN and NF on BRCA1 expression in the UACC3199 cell line, which was due in part to CpG demethylation of the *BRCA1* promoter [42]. In agreement with these data, we show here that GEN and GAL decrease CpG methylation at the *BRCA1* promoter in HCC38 cells. The dose of GEN (10 μM) used in this study was similar to the one required to CpG demethylate the *BRCA1* promoter in UACC3199 cells [42]. In humans, micromolar levels of GEN are achievable in the blood either through prolonged dietary exposure or supplementation. Serum levels of GEN in adults consuming a soy-rich Asian diet (~50 mg isoflavones/d) have been shown to approach concentrations of ~0.1–1.2 μM [75,76]. Moreover, in a phase I clinical trial administering GEN (600 mg/d) to post-menopausal women over an 84-d intervention, mean serum levels were ~11.1 μM, and in some subjects, levels reached >30 μM [77].

Several reports [28,29,30,31] support the capacity for GEN to demethylate tumor suppressor genes and reactivate their expression at concentrations similar to the one used here. In MCF7 (ERα-positive), MDA-MB-231 (TNBC), and MCF-10a (non-tumorigenic) cells, methylated DNA immunoprecipitation coupled to PCR amplification was used to determine that GEN (18.5 μM) treatment over 48 h demethylated both *BRCA1* and *BRCA2* [28]. In another study, methylation-sensitive restriction analysis (MRSA) showed that 10 μM GEN over a 96-h period decreased methylation and increased the expression of *RARβ* in both MC7 and MDA-MB-231 cells [29]. These dose and time points mimic those used in the current investigation to demethylate *BRCA1* in HCC38 cells. A relatively lower dose (3.125 μM) of GEN, administered over a six-day period was also shown to decrease methylation (determined by MSP) and increase the expression of *GSTP1* in MDA-MB-468 (TNBC) but not MCF7 cells [30]. Computational studies have demonstrated that the antagonistic effect of GEN against DNMT activity may be due to competitive binding with hemimethylated DNA at the catalytic site of DNMT1 [31].

A study by Xu and colleagues reported on the inability of the DNMT inhibitor 5-azacytidine to demethylate and restore the expression of *BRCA1* in HCC38 cells [45]. Thus, it is possible that the effect of GEN on *BRCA1* methylation and expression in HCC38 cells may not be due to the inhibition of DNMT1, but rather antagonism toward the AHR. Several lines of evidence support this speculation. First, the influence of GEN on *BRCA1* methylation and expression in HCC38 cells is analogous to that of known AHR antagonists, suggesting that the activation of *BRCA1* could be related to inhibitory effects on AHR binding/activity at the *BRCA1* promoter. Second, the treatment with GEN decreases the constitutive binding of AHR at *BRCA1* exon 1a, which is a response that is similar to the one elicited by the high-affinity AHR antagonists GAL and CH-223191. Third, in addition to antagonizing constitutively active AHR, both GEN and GAL prevent TCDD-induced binding of AHR at *BRCA1* exon 1a in MCF7 cells. This is consistent with our previous studies showing that GEN prevents TCDD-induced CpG methylation and the downregulation of *BRCA1* in MCF7 [42].

Previous investigations suggest that GEN is a weak AHR antagonist [half maximal inhibitory concentration (IC_50_) >50 μM]. However, in vitro gel mobility shift assays using cytosolic AHR from rat livers were used as opposed to whole cell model systems [48]. In murine hepatoma Hepa-1c1c7 cells, GEN dose-dependently inhibited (0.1–20 μg/mL) TCDD-mediated activation of an XRE-driven reporter system, and in human HepG2 cells, GEN (50 μM) repressed the basal and TCDD-dependent expression of *CYP1A1* [78]. In Caco2 colon cancer cells, GEN (50 μM) decreased nuclear AHR levels and prevented TCDD-induced AHR nuclear localization [50]. In MCF7 BC cells, GEN dose-dependently (1–20 μM) decreased the basal expression of *CYP1A1* and *CYP1B1* [79], but did not antagonize the TCDD-dependent (5 nM) activation of a reporter system at 1 and 10-μM doses [80]. Studies in T47D ER+ human BC cells indicate that GEN may be a partial agonist/antagonist in BC [51]. Alone, GEN (40 μM, max dose used in this study) was shown to act as a weak agonist, eliciting a reporter gene response <20% of that elicited by 10 nM TCDD. However, in T47D cells, 20 and 40-μM doses of GEN decreased the TCDD-mediated activation of the AHR-driven reporter system. This activity is similar to that of GAL in MCF7 cells. Alone, GAL increased *CYP1A1* expression, but attenuated TCDD-dependent and DMBA-dependent induction in co-treatment experiments [81]. Our data suggest that in BC cells with activated AHR (either constitutive or induced), GEN exerts antagonism toward AHR-dependent CpG methylation of *BRCA1*.

A significant clinical burden for TNBC patients is a lack of targeted therapeutics and reliance on systemic chemotherapy as the mainline neoadjuvant treatment option [55]. Approximately, only 20–30% of TBNC patients have a pathological complete response to neoadjuvant therapy [3]. The BRCA1 protein is a necessary factor for the transactivation of *ESR1.* Previous studies demonstrated that the transfection of ERα-positive BC cells with siRNA against *BRCA1* silenced the expression of *ESR1,* whereas the ectopic expression of a wild-type BRCA1 construct into the *BRCA1*-mutated/ERα-negative HCC1937 cell line rescued the ERα protein [52]. The latter outcome was shown to modulate the response of HCC1937 cells to the growth inhibitory effects of the antiestrogen fulvestrant. In the present study, we show that the upregulation of BRCA1 by GEN occurs in parallel with increased ERα expression. Moreover, cells pretreated with GEN were sensitized to the antiproliferative effects of 4-OHT, which is the active metabolite of the antiestrogen tamoxifen. Similar effects were observed with the control compound GAL. The capacity for GEN to sensitize TNBC cells to tamoxifen through ERα upregulation has been previously demonstrated in the MDA-MB-231 cell line, which is a model of TNBC [82]. Moreover, in rats, the dietary administration of GEN starting from PND15 was shown to improve the response of DMBA-induced mammary tumors to tamoxifen therapy [34]. Although previous studies in MDA-MB-231 cells linked this sensitization effect to decreased *ESR1* methylation [82], in the present study, we showed no difference in *ESR1* methylation between HCC38 and MCF7 cells. These results lend support to the possibility that *BRCA1* hypermethylation may drive an ERα-negative phenotype through loss of the BRCA1-dependent transactivation of *ESR1*, suggesting that compounds (i.e., GEN) that reduce *BRCA1* CpG methylation may hold promise as both preventive and adjuvant therapeutics for TNBC.

## 5. Conclusions

In summary, the current study provides in vivo evidence that lifetime exposure to dietary GEN, starting at conception, lowers CpG methylation at the *Brca1* gene in mammary glands of adult female mouse offspring. Our data suggest that the demethylating effects of GEN on *Brca1* are due to antagonism toward the AHR. This was demonstrated by a decrease in *Cyp1b1* expression, which is a putative AHR target, in adult mammary glands from mice exposed to dietary GEN through the lifetime. This conclusion was further supported by cell culture experiments in TNBC cells that overexpress a constitutively active AHR. These experiments documented the capacity for GEN to rescue *BRCA1* expression through demethylation of the *BRCA1* promoter. The latter effect was attributable to decreased binding of AHR at XRE sequences proximal to the *BRCA1* exon 1a. Finally, the GEN-dependent upregulation of *BRCA1* restored the expression of ERα, which imparted sensitivity to the antiestrogen 4-OHT. We conclude that dietary GEN may hold promise as a therapeutic agent for TNBC with activated AhR.

## References

[1] Bray F., Ferlay J., Soerjomataram I., Siegel R.L., Torre L.A., Jemal A. (2018). Global cancer statistics 2018: Globocan estimates of incidence and mortality worldwide for 36 cancers in 185 countries. CA Cancer J. Clin..

[2] Guarneri V., Dieci M.V., Conte P. (2013). Relapsed triple-negative breast cancer: Challenges and treatment strategies. Drugs.

[3] Nakashoji A., Matsui A., Nagayama A., Iwata Y., Sasahara M., Murata Y. (2017). Clinical predictors of pathological complete response to neoadjuvant chemotherapy in triple-negative breast cancer. Oncol. Lett..

[4] Savage K.I., Harkin D.P. (2015). Brca1, a ‘complex’ protein involved in the maintenance of genomic stability. FEBS J..

[5] Kuchenbaecker K.B., Hopper J.L., Barnes D.R., Phillips K.A., Mooij T.M., Roos-Blom M.J., Jervis S., van Leeuwen F.E., Milne R.L., Andrieu N. (2017). Risks of breast, ovarian, and contralateral breast cancer for brca1 and brca2 mutation carriers. Jama.

[6] Silver D.P., Richardson A.L., Eklund A.C., Wang Z.C., Szallasi Z., Li Q., Juul N., Leong C.O., Calogrias D., Buraimoh A. (2010). Efficacy of neoadjuvant cisplatin in triple-negative breast cancer. J. Clin. Oncol..

[7] Tian T., Shan L., Yang W., Zhou X., Shui R. (2019). Evaluation of the brcaness phenotype and its correlations with clinicopathological features in triple-negative breast cancers. Hum. Pathol..

[8] Zhang L., Long X. (2015). Association of brca1 promoter methylation with sporadic breast cancers: Evidence from 40 studies. Sci. Rep..

[9] Brianese R.C., Nakamura K.D.M., Almeida F., Ramalho R.F., Barros B.D.F., Ferreira E.N.E., Formiga M., de Andrade V.P., de Lima V.C.C., Carraro D.M. (2018). Brca1 deficiency is a recurrent event in early-onset triple-negative breast cancer: A comprehensive analysis of germline mutations and somatic promoter methylation. Breast Cancer Res. Treat..

[10] Lips E.H., Mulder L., Oonk A., van der Kolk L.E., Hogervorst F.B., Imholz A.L., Wesseling J., Rodenhuis S., Nederlof P.M. (2013). Triple-negative breast cancer: Brcaness and concordance of clinical features with brca1-mutation carriers. Br. J. Cancer.

[11] Ignatov T., Poehlmann A., Ignatov A., Schinlauer A., Costa S.D., Roessner A., Kalinski T., Bischoff J. (2013). Brca1 promoter methylation is a marker of better response to anthracycline-based therapy in sporadic tnbc. Breast Cancer Res. Treat..

[12] Esteller M., Fraga M.F., Guo M., Garcia-Foncillas J., Hedenfalk I., Godwin A.K., Trojan J., Vaurs-Barriere C., Bignon Y.J., Ramus S. (2001). DNA methylation patterns in hereditary human cancers mimic sporadic tumorigenesis. Hum. Mol. Genet..

[13] Van Heetvelde M., Van Bockstal M., Poppe B., Lambein K., Rosseel T., Atanesyan L., Deforce D., Van Den Berghe I., De Leeneer K., Van Dorpe J. (2018). Accurate detection and quantification of epigenetic and genetic second hits in brca1 and brca2-associated hereditary breast and ovarian cancer reveals multiple co-acting second hits. Cancer Lett..

[14] Vos S., Moelans C.B., van Diest P.J. (2017). Brca promoter methylation in sporadic versus brca germline mutation-related breast cancers. Breast Cancer Res..

[15] Rossi R.E., Pericleous M., Mandair D., Whyand T., Caplin M.E. (2014). The role of dietary factors in prevention and progression of breast cancer. Anticancer. Res..

[16] Tham D.G.C., Haskell W. (1998). Clinical review 97: Potential health benefits of dietary phytoestrogens: A review of the clinical, epidemiological, and mechanistic evidence. J. Clin. Endocrinol. Metab..

[17] Yang Z., Kulkarni K., Zhu W., Hu M. (2012). Bioavailability and pharmacokinetics of genistein: Mechanistic studies on its adme. Anticancer. Agents Med. Chem..

[18] Liu X.O., Huang Y.B., Gao Y., Chen C., Yan Y., Dai H.J., Song F.J., Wang Y.G., Wang P.S., Chen K.X. (2014). Association between dietary factors and breast cancer risk among chinese females: Systematic review and meta-analysis. Asian Pac. J. Cancer Prev..

[19] Qin L.Q., Xu J.Y., Wang P.Y., Hoshi K. (2006). Soyfood intake in the prevention of breast cancer risk in women: A meta-analysis of observational epidemiological studies. J. Nutr. Sci. Vitaminol..

[20] Trock B.J., Hilakivi-Clarke L., Clarke R. (2006). Meta-analysis of soy intake and breast cancer risk. J. Natl. Cancer Inst..

[21] Woo H.D., Park S., Oh K., Kim H.J., Shin H.R., Moon H.K., Kim J. (2014). Diet and cancer risk in the korean population: A meta-analysis. Asian Pac. J. Cancer Prev..

[22] Wu Y.C., Zheng D., Sun J.J., Zou Z.K., Ma Z.L. (2015). Meta-analysis of studies on breast cancer risk and diet in chinese women. Int J. Clin. Exp. Med..

[23] Chen M., Rao Y., Zheng Y., Wei S., Li Y., Guo T., Yin P. (2014). Association between soy isoflavone intake and breast cancer risk for pre- and post-menopausal women: A meta-analysis of epidemiological studies. PLoS ONE.

[24] Dong J.Y., Qin L.Q. (2011). Soy isoflavones consumption and risk of breast cancer incidence or recurrence: A meta-analysis of prospective studies. Breast Cancer Res. Treat..

[25] Russo M., Russo G.L., Daglia M., Kasi P.D., Ravi S., Nabavi S.F., Nabavi S.M. (2016). Understanding genistein in cancer: The “good” and the “bad” effects: A review. Food Chem..

[26] Jiang H., Fan J., Cheng L., Hu P., Liu R. (2018). The anticancer activity of genistein is increased in estrogen receptor beta 1-positive breast cancer cells. Onco Targets Ther..

[27] Fang Y., Zhang Q., Wang X., Yang X., Wang X., Huang Z., Jiao Y., Wang J. (2016). Quantitative phosphoproteomics reveals genistein as a modulator of cell cycle and DNA damage response pathways in triple-negative breast cancer cells. Int. J. Oncol..

[28] Bosviel R., Dumollard E., Dechelotte P., Bignon Y.J., Bernard-Gallon D. (2012). Can soy phytoestrogens decrease DNA methylation in brca1 and brca2 oncosuppressor genes in breast cancer?. Omics.

[29] Lubecka K., Kaufman-Szymczyk A., Cebula-Obrzut B., Smolewski P., Szemraj J., Fabianowska-Majewska K. (2018). Novel clofarabine-based combinations with polyphenols epigenetically reactivate retinoic acid receptor beta, inhibit cell growth, and induce apoptosis of breast cancer cells. Int. J. Mol. Sci.

[30] King-Batoon A., Leszczynska J.M., Klein C.B. (2008). Modulation of gene methylation by genistein or lycopene in breast cancer cells. Environ. Mol. Mutagen..

[31] Xie Q., Bai Q., Zou L.Y., Zhang Q.Y., Zhou Y., Chang H., Yi L., Zhu J.D., Mi M.T. (2014). Genistein inhibits DNA methylation and increases expression of tumor suppressor genes in human breast cancer cells. Genes Chromosomes Cancer.

[32] Luijten M., Thomsen A.R., van den Berg J.A., Wester P.W., Verhoef A., Nagelkerke N.J., Adlercreutz H., van Kranen H.J., Piersma A.H., Sorensen I.K. (2004). Effects of soy-derived isoflavones and a high-fat diet on spontaneous mammary tumor development in tg.Nk (mmtv/c-neu) mice. Nutr. Cancer.

[33] Thomsen A.R., Mortensen A., Breinholt V.M., Lindecrona R.H., Penalvo J.L., Sorensen I.K. (2005). Influence of prevastein, an isoflavone-rich soy product, on mammary gland development and tumorigenesis in tg.Nk (mmtv/c-neu) mice. Nutr. Cancer.

[34] Zhang X., Cook K.L., Warri A., Cruz I.M., Rosim M., Riskin J., Helferich W., Doerge D., Clarke R., Hilakivi-Clarke L. (2017). Lifetime genistein intake increases the response of mammary tumors to tamoxifen in rats. Clin. Cancer Res..

[35] De Assis S., Warri A., Benitez C., Helferich W., Hilakivi-Clarke L. (2011). Protective effects of prepubertal genistein exposure on mammary tumorigenesis are dependent on brca1 expression. Cancer Prev. Res. (Phila).

[36] Hockings J.K., Thorne P.A., Kemp M.Q., Morgan S.S., Selmin O., Romagnolo D.F. (2006). The ligand status of the aromatic hydrocarbon receptor modulates transcriptional activation of brca-1 promoter by estrogen. Cancer Res..

[37] Tian J., Feng Y., Fu H., Xie H.Q., Jiang J.X., Zhao B. (2015). The aryl hydrocarbon receptor: A key bridging molecule of external and internal chemical signals. Environ. Sci. Technol..

[38] Yueh M.F., Huang Y.H., Hiller A., Chen S., Nguyen N., Tukey R.H. (2003). Involvement of the xenobiotic response element (xre) in ah receptor-mediated induction of human udp-glucuronosyltransferase 1a1. J. Biol. Chem..

[39] Jeffy B.D., Hockings J.K., Kemp M.Q., Morgan S.S., Hager J.A., Beliakoff J., Whitesell L.J., Bowden G.T., Romagnolo D.F. (2005). An estrogen receptor-alpha/p300 complex activates the brca-1 promoter at an ap-1 site that binds jun/fos transcription factors: Repressive effects of p53 on brca-1 transcription. Neoplasia.

[40] Papoutsis A.J., Borg J.L., Selmin O.I., Romagnolo D.F. (2012). Brca-1 promoter hypermethylation and silencing induced by the aromatic hydrocarbon receptor-ligand tcdd are prevented by resveratrol in mcf-7 cells. J. Nutr. Biochem..

[41] Papoutsis A.J., Lamore S.D., Wondrak G.T., Selmin O.I., Romagnolo D.F. (2010). Resveratrol prevents epigenetic silencing of brca-1 by the aromatic hydrocarbon receptor in human breast cancer cells. J. Nutr..

[42] Romagnolo D.F., Donovan M.G., Papoutsis A.J., Doetschman T.C., Selmin O.I. (2017). Genistein prevents *brca1* cpg methylation and proliferation in human breast cancer cells with activated aromatic hydrocarbon receptor. Curr. Dev. Nutr..

[43] Livak K.J., Schmittgen T.D. (2001). Analysis of relative gene expression data using real-time quantitative pcr and the 2(-delta delta c(t)) method. Methods.

[44] Rice J.C., Massey-Brown K.S., Futscher B.W. (1998). Aberrant methylation of the brca1 cpg island promoter is associated with decreased brca1 mrna in sporadic breast cancer cells. Oncogene.

[45] Xu J., Huo D., Chen Y., Nwachukwu C., Collins C., Rowell J., Slamon D.J., Olopade O.I. (2010). Cpg island methylation affects accessibility of the proximal brca1 promoter to transcription factors. Breast Cancer Res. Treat..

[46] Romagnolo D.F., Papoutsis A.J., Laukaitis C., Selmin O.I. (2015). Constitutive expression of ahr and brca-1 promoter cpg hypermethylation as biomarkers of erα-negative breast tumorigenesis. BMC Cancer.

[47] Yang X., Solomon S., Fraser L.R., Trombino A.F., Liu D., Sonenshein G.E., Hestermann E.V., Sherr D.H. (2008). Constitutive regulation of cyp1b1 by the aryl hydrocarbon receptor (ahr) in pre-malignant and malignant mammary tissue. J. Cell. Biochem..

[48] Ashida H., Fukuda I., Yamashita T., Kanazawa K. (2000). Flavones and flavonols at dietary levels inhibit a transformation of aryl hydrocarbon receptor induced by dioxin. FEBS Lett..

[49] Tomblin J.K., Arthur S., Primerano D.A., Chaudhry A.R., Fan J., Denvir J., Salisbury T.B. (2016). Aryl hydrocarbon receptor (ahr) regulation of l-type amino acid transporter 1 (lat-1) expression in mcf-7 and mda-mb-231 breast cancer cells. Biochem. Pharmacol..

[50] Kasai S., Kikuchi H. (2010). The inhibitory mechanisms of the tyrosine kinase inhibitors herbimycin a, genistein, and tyrphostin b48 with regard to the function of the aryl hydrocarbon receptor in caco-2 cells. Biosci. Biotechnol. Biochem..

[51] Van der Heiden E., Bechoux N., Muller M., Sergent T., Schneider Y.J., Larondelle Y., Maghuin-Rogister G., Scippo M.L. (2009). Food flavonoid aryl hydrocarbon receptor-mediated agonistic/antagonistic/synergic activities in human and rat reporter gene assays. Anal. Chim. Acta.

[52] Hosey A.M., Gorski J.J., Murray M.M., Quinn J.E., Chung W.Y., Stewart G.E., James C.R., Farragher S.M., Mulligan J.M., Scott A.N. (2007). Molecular basis for estrogen receptor alpha deficiency in brca1-linked breast cancer. J. Natl. Cancer Inst..

[53] Dent R., Hanna W.M., Trudeau M., Rawlinson E., Sun P., Narod S.A. (2009). Pattern of metastatic spread in triple-negative breast cancer. Breast Cancer Res. Treat..

[54] Rakha E.A., Chan S. (2011). Metastatic triple-negative breast cancer. Clin. Oncol (R Coll Radiol).

[55] Schmadeka R., Harmon B.E., Singh M. (2014). Triple-negative breast carcinoma: Current and emerging concepts. Am. J. Clin. Pathol..

[56] Vacher S., Castagnet P., Chemlali W., Lallemand F., Meseure D., Pocard M., Bieche I., Perrot-Applanat M. (2018). High ahr expression in breast tumors correlates with expression of genes from several signaling pathways namely inflammation and endogenous tryptophan metabolism. PLoS ONE.

[57] Trombino A.F., Near R.I., Matulka R.A., Yang S., Hafer L.J., Toselli P.A., Kim D.W., Rogers A.E., Sonenshein G.E., Sherr D.H. (2000). Expression of the aryl hydrocarbon receptor/transcription factor (ahr) and ahr-regulated cyp1 gene transcripts in a rat model of mammary tumorigenesis. Breast Cancer Res. Treat..

[58] Eltom S.E., Gasmelseed A.A., Saudoudi-Guentri D. (2006). The aryl hydrocarbon receptor is over-expressed and constitutively activated in advanced breast carcinoma. Cancer Res..

[59] Li Z.D., Wang K., Yang X.W., Zhuang Z.G., Wang J.J., Tong X.W. (2014). Expression of aryl hydrocarbon receptor in relation to p53 status and clinicopathological parameters in breast cancer. Int. J. Clin. Exp. Pathol..

[60] Saito R., Miki Y., Hata S., Takagi K., Iida S., Oba Y., Ono K., Ishida T., Suzuki T., Ohuchi N. (2014). Aryl hydrocarbon receptor in breast cancer-a newly defined prognostic marker. Horm. Cancer.

[61] Brooks J., Eltom S.E. (2011). Malignant transformation of mammary epithelial cells by ectopic overexpression of the aryl hydrocarbon receptor. Curr. Cancer Drug Targets.

[62] Goode G., Ballard B.R., Manning H.C., Freeman M.L., Kang Y., Eltom S.E. (2013). Knockdown of aberrantly upregulated aryl hydrocarbon receptor reduces tumor growth and metastasis of mda-mb-231 human breast cancer cell line. Int. J. Cancer.

[63] Andlauer W., Kolb J., Stehle P., Furst P. (2000). Absorption and metabolism of genistein in isolated rat small intestine. J. Nutr..

[64] Andrade J.E., Twaddle N.C., Helferich W.G., Doerge D.R. (2010). Absolute bioavailability of isoflavones from soy protein isolate-containing food in female balb/c mice. J. Agric. Food Chem..

[65] Chen J., Wang S., Jia X., Bajimaya S., Lin H., Tam V.H., Hu M. (2005). Disposition of flavonoids via recycling: Comparison of intestinal versus hepatic disposition. Drug Metab. Dispos..

[66] Coldham N.G., Sauer M.J. (2000). Pharmacokinetics of [(14)c]genistein in the rat: Gender-related differences, potential mechanisms of biological action, and implications for human health. Toxicol. Appl. Pharmacol..

[67] Coldham N.G., Zhang A.Q., Key P., Sauer M.J. (2002). Absolute bioavailability of [14c] genistein in the rat; plasma pharmacokinetics of parent compound, genistein glucuronide and total radioactivity. Eur J. Drug Metab. Pharmacokinet..

[68] Hosoda K., Furuta T., Yokokawa A., Ogura K., Hiratsuka A., Ishii K. (2008). Plasma profiling of intact isoflavone metabolites by high-performance liquid chromatography and mass spectrometric identification of flavone glycosides daidzin and genistin in human plasma after administration of kinako. Drug Metab. Dispos..

[69] Yang Z., Zhu W., Gao S., Xu H., Wu B., Kulkarni K., Singh R., Tang L., Hu M. (2010). Simultaneous determination of genistein and its four phase ii metabolites in blood by a sensitive and robust uplc-ms/ms method: Application to an oral bioavailability study of genistein in mice. J. Pharm. Biomed. Anal..

[70] Hosoda K., Furuta T., Yokokawa A., Ishii K. (2010). Identification and quantification of daidzein-7-glucuronide-4′-sulfate, genistein-7-glucuronide-4′-sulfate and genistein-4′,7-diglucuronide as major metabolites in human plasma after administration of kinako. Anal. Bioanal. Chem..

[71] Chang H.C., Churchwell M.I., Delclos K.B., Newbold R.R., Doerge D.R. (2000). Mass spectrometric determination of genistein tissue distribution in diet-exposed sprague-dawley rats. J. Nutr..

[72] Warri A., Saarinen N.M., Makela S., Hilakivi-Clarke L. (2008). The role of early life genistein exposures in modifying breast cancer risk. Br. J. Cancer.

[73] Novikov O., Wang Z., Stanford E.A., Parks A.J., Ramirez-Cardenas A., Landesman E., Laklouk I., Sarita-Reyes C., Gusenleitner D., Li A. (2016). An aryl hydrocarbon receptor-mediated amplification loop that enforces cell migration in er(−)/pr(−)/her2(−) human breast cancer cells. Mol. Pharmacol..

[74] Vogel C.F.A., Haarmann-Stemmann T. (2017). The aryl hydrocarbon receptor repressor - more than a simple feedback inhibitor of ahr signaling: Clues for its role in inflammation and cancer. Curr. Opin. Toxicol..

[75] Holder C.L., Churchwell M.I., Doerge D.R. (1999). Quantification of soy isoflavones, genistein and daidzein, and conjugates in rat blood using lc/es-ms. J. Agric. Food Chem..

[76] Adlercreutz H., Fotsis T., Watanabe S., Lampe J., Wahala K., Makela T., Hase T. (1994). Determination of lignans and isoflavonoids in plasma by isotope dilution gas chromatography-mass spectrometry. Cancer Detect. Prev..

[77] Pop E.A., Fischer L.M., Coan A.D., Gitzinger M., Nakamura J., Zeisel S.H. (2008). Effects of a high daily dose of soy isoflavones on DNA damage, apoptosis and estrogenic outcomes in healthy, postmenopausal women—A phase i clinical trial. Menopause.

[78] Kasai A., Hiramatsu N., Hayakawa K., Yao J., Kitamura M. (2008). Blockade of the dioxin pathway by herbal medicine formula bupleuri minor: Identification of active entities for suppression of ahr activation. Biol. Pharm. Bull..

[79] Dunlap T.L., Howell C.E., Mukand N., Chen S.N., Pauli G.F., Dietz B.M., Bolton J.L. (2017). Red clover aryl hydrocarbon receptor (ahr) and estrogen receptor (er) agonists enhance genotoxic estrogen metabolism. Chem. Res. Toxicol..

[80] Zhang S., Qin C., Safe S.H. (2003). Flavonoids as aryl hydrocarbon receptor agonists/antagonists: Effects of structure and cell context. Environ. Health Perspect..

[81] Ciolino H.P., Yeh G.C. (1999). The flavonoid galangin is an inhibitor of cyp1a1 activity and an agonist/antagonist of the aryl hydrocarbon receptor. Br. J. Cancer.

[82] Li Y., Meeran S.M., Patel S.N., Chen H., Hardy T.M., Tollefsbol T.O. (2013). Epigenetic reactivation of estrogen receptor-alpha (eralpha) by genistein enhances hormonal therapy sensitivity in eralpha-negative breast cancer. Mol. Cancer.

